# Slight up‐regulation of Kir2.1 channel promotes endothelial progenitor cells to transdifferentiate into a pericyte phenotype by Akt/mTOR/Snail pathway

**DOI:** 10.1111/jcmm.16944

**Published:** 2021-09-30

**Authors:** Xiaodong Cui, Xiaoxia Li, Yanting He, Jie Yu, Naijun Dong, Robert Chunhua Zhao

**Affiliations:** ^1^ Department of Basic Medicine Institute of Stem Cell and Regenerative Medicine Qingdao University Medical College Qingdao University Qingdao China; ^2^ School of Basic Medicine Sciences Weifang Medical University Weifang China

**Keywords:** differentiation, endothelial cells, endothelial progenitor cells, endothelial‐mesenchymal transition, pericytes

## Abstract

It was shown that endothelial progenitor cells (EPCs) have bidirectional differentiation potential and thus perform different biological functions. The purpose of this study was to investigate the effects of slight up‐regulation of the Kir2.1 channel on EPC transdifferentiation and the potential mechanism on cell function and transformed cell type. First, we found that the slight up‐regulation of Kir2.1 expression promoted the expression of the stem cell stemness factors ZFX and NS and inhibited the expression of senescence‐associated β‐galactosidase. Further studies showed the slightly increased expression of Kir2.1 could also improve the expression of pericyte molecular markers NG2, PDGFRβ and Desmin. Moreover, adenovirus‐mediated Kir2.1 overexpression had an enhanced contractile response to norepinephrine of EPCs. These results suggest that the up‐regulated expression of the Kir2.1 channel promotes EPC transdifferentiation into a pericyte phenotype. Furthermore, the mechanism of EPC transdifferentiation to mesenchymal cells (pericytes) was found to be closely related to the channel functional activity of Kir2.1 and revealed that this channel could promote EPC EndoMT by activating the Akt/mTOR/Snail signalling pathway. Overall, this study suggested that in the early stage of inflammatory response, regulating the Kir2.1 channel expression affects the biological function of EPCs, thereby determining the maturation and stability of neovascularization.

## 1 INTRODUCTION

Bone marrow–derived endothelial progenitor cells (EPCs) can be mobilized, homed to the site of local vascular injury and differentiated into vascular endothelial cells in vascular repair. Therefore, EPCs are the critical cells of postnatal neovascularization and angiogenesis. Accumulating evidence and our previous reports have shown the potential of treatment with EPC transplantation in animal models of carotid artery injury and hind limb and myocardial ischaemia, paving the way for clinical research.^1^, ^2^, ^3^, ^4^

However, an endothelial cell phenotype is not always the final endpoint of the differentiation of EPCs, which is not preset. The recent literature has shown that under certain physiological or pathophysiological conditions, EPCs can undergo an endothelial‐mesenchymal transition (EndoMT); thus, their function will presumably be altered.^5^, ^6^ Unfortunately, the underlying mechanism of this transformation process has not yet been clarified.

According to their functional characteristics and amino acid sequences, channels in the inward rectifier potassium channels (Kir) family can be divided into the following seven subfamilies into four functional groups: Kir1.x, Kir4.x, Kir5.x and Kir7.x (K^+^ transport channels); Kir2.x (classic K^+^ channels); Kir3.x (G‐protein‐gated K^+^ channels); and Kir6.x (ATP‐sensitive K^+^ channels).^7^ The channels in this family conduct more inward current under a negative voltage/balanced potential than under a negative current/outward voltage. Therefore, the Kir channels play an important role in maintaining the resting membrane potential (RMP) in most cells. However, the distributions of Kir subtypes vary due to cell heterogeneity, which also determines the differences in their biological functions. The roles of Kir channels in Andersen's syndrome, cardiac arrhythmias and hypokalaemic periodic paralysis, which have been extensively discussed, involve and affect cell migration, contraction and differentiation.^8^

Previous studies have shown that the Kir2.1 channel is the main inward rectifying potassium channel on EPCs.^9^ Our recent research also showed that the Kir2.1 channel is closely related to the differentiation of EPCs.^2^ Furthermore, blockade of the Kir2.1 channel was found to cause the depolarization of EPCs and accelerate the process of endothelial differentiation, which is related to the formation of autophagy.^2^ As shown by our further study, interestingly, we found that the expression of Kir2.1 was increased in EPCs due to oxidative stress caused by hydrogen peroxide. In contrast, inhibiting the channel function with the specific blocker ML133 could promote the occurrence of cell apoptosis (Figure S1). However, whether alterations in Kir2.1 channel expression affect the differentiation (transdifferentiation) or subsequent cytological functions of EPCs has not been discussed.

Here, we report that the slight up‐regulation of Kir2.1 promoted channel hyperpolarization, contributing to EPC mesenchymal transition, and that this process has an essential function in angiogenesis and vascular stability.

## 2 MATERIALS AND METHODS

### 2.1 Isolation, extraction, identification and culture of EPCs from rat bone marrow

Sprague Dawley rats weighing 200–300 g (Pengyue Laboratory Animal Breeding Company, Jinan, China) were euthanized by neck dislocation and then soaked in 70–75% alcohol for sterilization. The obtained bone marrow mononuclear cells were inoculated in a T25 culture bottle pre‐coated with fibronectin at 5 µg/cm^2^ and cultured in EGM‐2MV medium. The exact method was carried out by referring to our previous literature.^10^ Unless specified, cells at passage 3–4 were selected as the research object in this study. This study was carried out following the Helsinki Declaration and supervised and approved by the Review Committee for the Use of Human or Animal Subjects of Qingdao University and Weifang Medical University.

### 2.2 Chemicals and reagents

ML133 (Sigma‐Aldrich), MK2206 and RAD001 (MedChemExpress, USA) were dissolved in DMSO at working concentrations of 20, 5 and 10 nM, respectively. In addition, zacopride (Tocris Bioscience) was dissolved in pure water, and bacteria were removed with 0.22‐µm filter membranes.

### 2.3 Cell viability assay

EPC viability assay was performed by the Cell Counting Kit‐8 (CCK8) method. Specifically, cells were inoculated in 96‐well plates at a density of 5 × 10^3^/well. After 3 days of transfection with the adenovirus vector, the culture medium in each well was exchanged with 10 µl of CCK8 solution in 100 µl of culture solution. After 2 h, the optical density at 450 nm was detected with a microplate spectrophotometer.

### 2.4 Recombinant adenovirus or siRNA transfection

Adenovirus containing the rat Kir2.1 gene (NM_017296.1, GFP‐Ad‐Kir2.1 or Ad‐Kir2.1) was commercially designed and synthesized by HanBio Company (HanBio). In brief, the cells were washed twice with 1 × PBS, and serum‐free medium containing viral particles at different titres (MOIs) was slowly added to each well. Both Stealth RNAi^TM^ siRNA against rat Snail and negative control siRNA were purchased from Invitrogen (Thermal Fisher) The sequence of the siRNA was as follows: 5′‐AAUAUUUGCAGUUGAAGGCCUUCCG‐3′. Transfection with siRNA or scramble siRNA was carried out using Lipofectamine 3000 according to the manufacturer's recommended instructions.

### 2.5 SYBR Green–based quantitative RT‐PCR

Total mRNA was extracted from samples with TRIzol™ reagent (Invitrogen, Thermal Science). Reverse transcription and quantitative PCR were performed using TaKaRa One‐Step TB Green^®^ PrimeScript™, and the PCR samples were 25 µl. The reaction conditions were as follows: pre‐denaturation at 95°C for 30 s followed by 40 cycles of 95°C for 20 s and 60°C for 20 s. The following primer sequences were used: ZFX (XM_006257030.3) forward primer: 5′‐CTCTGACCGCTGATGTTGTTTC‐3′, reverse primer: 5′‐GTCCTCACAGTTGGCTTTGTCTT‐3′; nucleostemin (NS) (NM_175580.2) forward primer: 5′‐GAAATTAGCCCTGATGATGAGCAA‐3′, reverse primer: 5′‐TGAGGACACCTGCAACCAAGA‐3′; NG2 (NM_031022.1) forward primer: 5′‐AGCCCATGGCCTTCACTATCAC‐3′, reverse primer:. 5′‐CCGGCCCTGAATCACTGTCTA‐3′; and PDGFRβ (NM_031525.1) forward primer: 5′‐CCAGCACTGAGCTCTACAGCAA‐3′, reverse primer: 5′‐TGTCCAACATGGGCACGTAA‐3′. Transforming growth factor β1 (TGFβ1) (NM_021578.2) forward primer: 5′‐ GACCGCAACAACGCAATCTATGAC‐3′, 5′‐ CTGGCACTGCTTCCCGAATGTC‐3′. The sequences of the primers for the Kir2.1, CD31, vWF and GADPH genes are available in our previous study.^2^

### 2.6 Protein extractions and Western blot analysis

Detailed methods are available to our previous literature.^11^, ^12^ The protein antibodies used were as follows: antibodies against p53, SIRT1, NG2 and PDGFRβ were purchased from Abcam Company, and antibodies against p‐Akt, p‐mTOR and Snail were purchased from Cell Signal Company. Protein bands were visualized using a chemiluminescence and spectral fluorescence imaging system (Uvitec alliance Q9). The relative optical densities of the protein bands were analyzed with ImageJ v1.53c software.

### 2.7 Fluorescence‐activated cell sorting analysis

The cells were digested with a trypsin solution (0.25%) to obtain a cell suspension and then centrifuged at 500 × g for 10 min to obtain precipitation. CD31 (1:200; Millipore), CD29 (1:100; Cyagen), CD44 (1:100; Cyagen), vWF (1:200; Abcam) and CD45 (1:100; Bioss,) antibodies were incubated with the sample at 37°C for 1 h and fluorescent (PE or CY5, 1:200; Bioss, China) labelled secondary antibodies were stained at 4°C for 30 min before detection by FACS. Apoptosis was detected with 7‐AAD and Annexin V‐PE apoptosis detection kit (BD Biosciences) following the manufacturer's instructions.

### 2.8 Cell immunofluorescence assay

Cells were plated in 24‐well plates and fixed using 4% paraformaldehyde. After permeabilization with 0.1% Triton X‐100 at different times, anti‐Kir2.1(1:100; Abcam, USA, 3 min), anti‐NG2 (1:100; Abcam, non‐permeable cell treatment), anti‐PDGFRβ (1:100; Abcam, non‐permeable cell treatment), anti‐vWF (1:100; Proteintech, 5 min), anti‐Desmin antibody (1:200; Abcam) and anti‐CD31 (1:200; Abcam, 5 min) were added to the well and incubated at 4°C overnight. The cells were incubated with a fluorescence‐labelled secondary antibody for 1 h.

The RMP was detected by DiBAC4(3) (5 µM, US Everbright, China) staining. In brief, cells were incubated in culture medium containing DiBAC4(3) for 30 min and then washed twice with PBS.

The fluorescence intensity of protein expression or the cellular RMP was visualized and quantitatively analyzed by fluorescence microscopy (Olympus IX71, Japan) or confocal laser microscope (Leica TCS SP8, Germany).

### 2.9 ELISA

The level of TGFβ1 in the supernatant was determined by ELISA according to the manufacturer's instructions (Sangon Biotechnology). The optical density at 450 nm was measured using Thermo Fisher software (USA).

### 2.10 Cellular senescence detection (senescence‐associated β‐galactosidase staining)

Cell senescence was detected using a senescence‐associated β‐galactosidase (SA‐β‐Gal) staining kit (Beyotime, China) following the manufacturer's instructions. In brief, the cell culture solution in the 6‐well plate was removed, the cells were washed with PBS three times, and 1 ml of β‐Gal fixation solution was added and incubated for 15 min at room temperature to fix the cells. Then, the cells were washed with PBS, and 1 ml of a staining solution containing β‐Gal stain A\B\C and X‐Gal solution was added to each well. Cells were incubated overnight at 37°C and photographed under an optical microscope the next day. Cells expressing blue β‐Gal were positive, and the corresponding senescence rate was calculated.

### 2.11 In vitro coculture Matrigel angiogenesis assay

First, 100 µl of Matrigel (BD Biosciences, USA) was quickly placed in a 96‐well plate on ice and then incubated at 37°C for 10 min. Then, EPCs transfected with pAV‐GFP‐Kir2.1 and CM‐Dil‐labelled rat aortic endothelial cells (RAECs) (provided by UbiGene Company, China) were mixed at a ratio of 1:1, and the cell mixture was added to an equal amount of Matrigel matrix. Tube formation and stability were observed and analyzed under a fluorescence microscope at 6 and 24 h. ImageJ software was used to calculate the length of tubular structures and the maintenance rate of capillary structures.

### 2.12 Cell contraction assay

Noradrenaline (NE) (0.1 mM, Grand Pharma Company) dissolved in cell culture medium was used as a stimulant. GFP‐labelled EPCs were treated for 1 h, and the degree of cell contraction was observed under a fluorescence microscope. Cell morphology was measured before and after NE intervention and compared with that in the control group.

The collagen contraction model was performed by the company's kit instructions (Cell BioLabs). In detail, the collagen gel working solution is configured on ice that containing the collagen fluid, 5 × DMEM and neutralization solution. Then, the target cells were digested, and the cell concentration was adjusted to 3 × 10^6^/ml of culture medium. A total of 0.5 ml of the cell‐collagen mixture containing 1 part cell suspension and 4 parts the collagen working solution was added into per well in a 24‐well plate and incubated for 1 h in a cell incubator in order to promote the collagen polymerization. At last, 1.0 ml of culture medium was added atop each lattice. After 48 h of incubation, the tensioned collagen matrix was released with 10‐μl sterile pipette tips before the NE working solution was intervened. The gel area changes were measured at various times with ImageJ v1.53c software.

### 2.13 Statistical analysis

Experimental data are represented as the mean ± SD. Student's *t* test was used to analyze data between two groups. Data among the three groups were analyzed by one‐way ANOVA (Prism GraphPad 8.2.1). Differences with *p* ≤ 0.05 were considered statistically significant.

## 3 RESULTS

### 3.1 The slightly up‐regulated expression of Kir2.1 could promote the maintenance of stemness and reduce the senescence of EPCs

In our previous study, we found that blocking the function of Kir2.1 or knocking out its expression could accelerate the differentiation of EPCs into endothelial cells.^2^ In addition, the expression of Kir2.1 was found to be slightly increased when EPCs were exposed to temporary oxidative stress (various concentrations of H_2_O_2_ stress, Figure S1A), but apoptosis was significantly enhanced by the specific Kir2.1 channel blocker ML133 in a lower concentration of H_2_O_2_ (Figure S1B).

**FIGURE 1:**
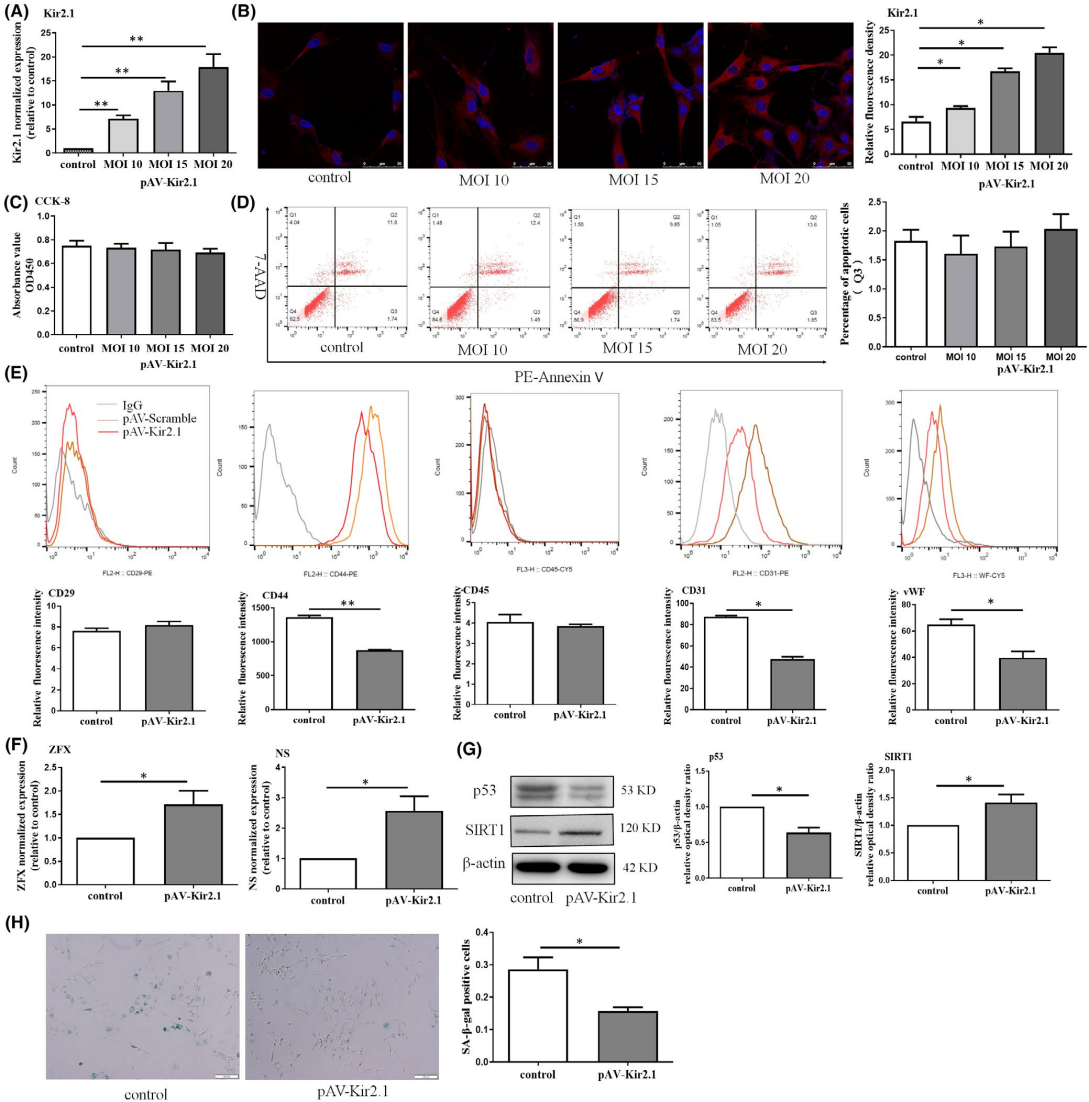
Slightly up‐regulated expression of Kir2.1 can promote the maintenance of stemness and reduce the senescence of EPCs. (A) Real‐time RT‐PCR detected Kir2.1 expression. (B) Kir2.1 protein expression was detected by cell immunofluorescence. Relative data are used to represent the Kir2.1 gene or protein expression in EPCs transfected with adenovirus at different MOIs. (C) Cell viability in cells transfected with adenovirus at different MOIs was detected by the CCK8 assay. (D) Effect of the transfection of Kir2.1 with pAV‐scramble on cell apoptosis determined by 7‐AAD/PE‐Annexin V staining. (E) The marker molecules were CD29, CD45, CD44, CD31 and vWF were detected by FACS. (F) The stem cell stemness genes ZFX and NS in EPCs transfected with pAV‐Kir2.1 were detected by real‐time RT‐PCR. (G) Expression of the senescence‐related protein p53 and SIRT1 in EPCs was detected by WB analysis. (H) EPC senescence was detected using the SA‐β‐Gal method. (*p < 0.05 and **p < 0.01). All values represent the mean ± SD for three separate experiments. Scale bars in (B) represent 50 μm, while those in (G) represent 100 μm

Therefore, EPCs were transfected by adenovirus containing the Kir2.1 gene with different MOI values to promote different degrees of expression. First, the expression of Kir2.1 was slightly increased when adenovirus was transfected at an MOI no higher than 20 (Figure 1A and B, Figure S2). Second, compared with the control group, the MOI (10, 15 and 20) used in viral transfection did not affect EPC activity (Figure 1C) or the occurrence of apoptosis (Figure 1D). In addition, compared with the control group (0 µM), the apoptosis of EPCs treated with slight hydrogen peroxide (12.5 µM) was not significantly changed. However, the expression of Kir2.1 was slightly increased (about 1.98 times than the control group). Moreover, ML133, the functional blocker of Kir2.1, could aggravate the apoptosis of hydrogen peroxide–induced EPCs. So combined with the supplementary data results (Figure S1 and 2), MOI = 15 was selected in the following research and we define the up‐regulation of Kir2.1 protein expression as ‘slight‐up’.

Next, after Kir2.1 transfection, the population of EPCs was performed by FACS as shown in Figure 1E. Compared with the control group, the expression of CD44, CD31 and vWF was decreased, but there was no change in CD29 and CD45 expression. Stem and progenitor cells are characterized by their self‐renewal and ability to maintain stemness. In the case of EPCs, their stemness and senescence determine their capacity to accumulate for postnatal vascular repair. Hamid et al.^13^ showed that ZFX and NS were marker molecules reflecting the stemness or self‐renewal ability of EPCs, while other molecules, such as OCT4 and NANOG, had low or no expression, which is consistent with our results (Figure S3). In this study, we found that slightly increased Kir2.1 expression was shown to promote the expression of ZFX and NS (Figure 1F), suggesting that EPC stemness maintenance was enhanced. In addition, increased expression of Kir2.1 reduced the expression of p53 but slightly promoted the expression of SIRT1, which indicates that Kir2.1 can delay or inhibit the ageing of EPCs (Figure 1G). Furthermore, this effect was confirmed by SA‐β‐Gal staining (Figure 1H).

### 3.2 The slightly up‐regulated expression of Kir2.1 could promote EPC transdifferentiation into a pericyte phenotype

**FIGURE 2:**
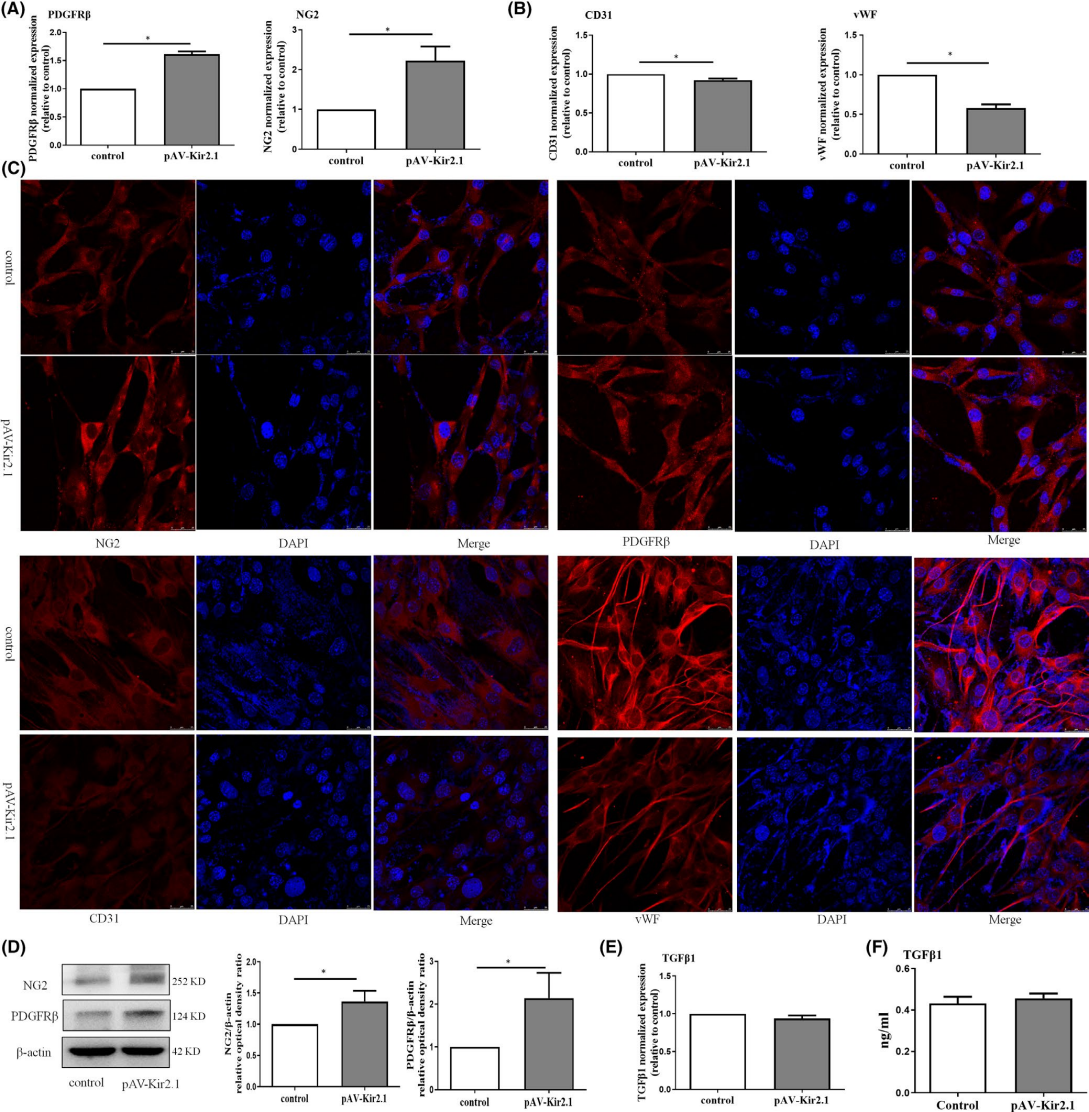
Slightly up‐regulated expression of Kir2.1 can promote EPC transdifferentiation into a pericyte phenotype. Expression of the pericyte markers NG2 and PDGFRβ (A) and expression of the endothelial cell markers CD31 and vWF (B) were detected by real‐time RT‐PCR. Data represent the relative value compared with the control group. (C) Endothelial cell marker molecules CD31 and vWF, pericyte marker NG2 and PDGFRβ, were performed by double immunofluorescence staining with antibodies and DAPI solution. The fluorescence intensity or distribution of protein expression was visualized by confocal laser microscope. (D) Expression of the pericyte markers NG2 and PDGFRβ was detected by WB analysis. (E) The expression of TGF‐β1 was detected by real‐time RT‐PCR. (F) The level of TGF‐β1 in the supernatant of culture medium after EPCs transfection was detected by ELISA methods. Data represent the relative value compared with the control group. All values represent the mean ± SD for three separate experiments. (*p < 0.05). Scale bars in (C) represent 25 μm

Under a moderate oxidative stress environment, neovascularization is not decreased but rather increased.^14^, ^15^ Unfortunately, the mechanism of neovascularization is not clear. We found that Kir2.1 overexpression through transfection with adenovirus could promote expression of the pericyte markers NG2 and PDGFRβ (Figure 2AandD). In contrast, the data showed that the gene and protein expression of the endothelial marker molecules CD31 and vWF was decreased (Figure 2B and Figure 1E), indicating that Kir2.1 overexpression inhibited EPC differentiation into endothelial cells. Moreover, to improve the association between Kir2.1 overexpression and EndoMT, endothelial cell marker molecules CD31 and vWF, pericyte marker NG2, and PDGFRβ, were performed by double immunofluorescence staining with antibodies and DAPI solution (Figure 2C). In addition, supplementary data 4 showed triple‐cell fluorescence staining including the green‐staining of Kir2.1 protein (Figure S4A). Studies have shown that TGFβ1 may play an essential role in introducing mesenchymal transdifferentiation of endothelial cells or endothelial progenitor cells.^16^, ^17^ However, our results did not show any change in EPCs TGFβ1 expression caused by Kir2.1 alteration (Figure 2EandF).

### 3.3 EPC‐derived pericytes express the pericyte marker protein Desmin and promote the maturation and stabilization of neovascularization

**FIGURE 3:**
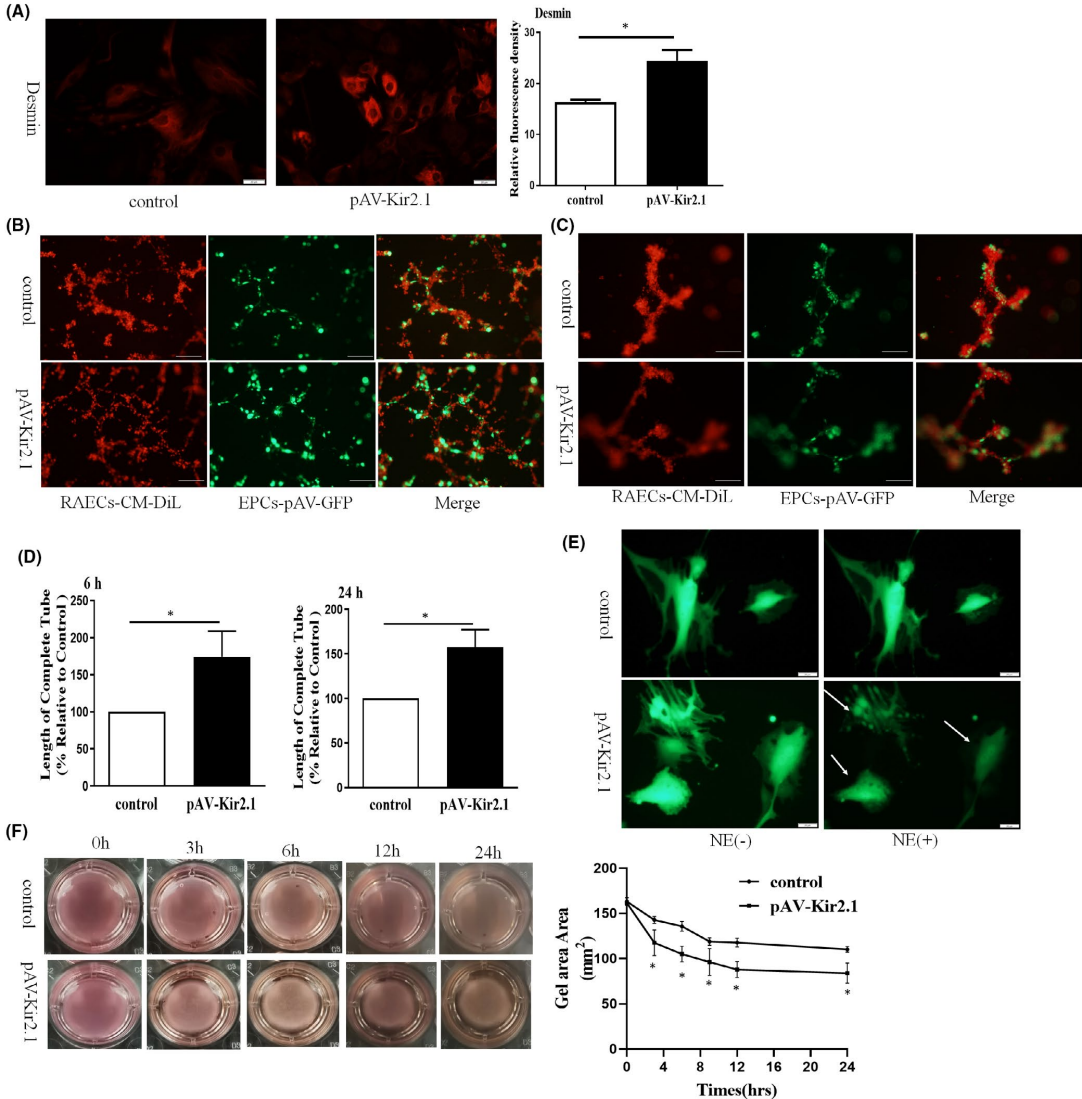
EPC‐derived pericytes express the pericyte marker protein Desmin and promote the maturation of neovascularization. (A) Expression of the pericyte function‐related protein Desmin was detected by cell immunofluorescence. Relevant data are used to represent the protein expression on EPCs. (B‐C) Matrigel angiogenesis assay based on RAECs (CM‐Dil‐labelled) and pAV‐GFP‐Kir2.1‐EPCs (GFP) coculture. Tube formation was observed and analyzed under a fluorescence microscope at 6 (B) and 24 h (C). (D) ImageJ software was used to calculate the length of tubular structures of capillary‐like structures. Relevant experimental data compared with the control group. (E) GFP‐labelling‐EPCs were stimulated by NE (0.1 mM), and cell contraction was qualitatively determined by cell fluorescence microscopy. (F) Cells were incorporated into the collagen gel, and the gel stress was released before the cells were treated with NE (0.1 mM). The alters of per well gel area in a 24‐well plate were measured by ImageJ at different points in time. All values represent the mean±SD for 3 separate experiments. Scale bars in (A) represent 20 μm, (B) 100 μm, while those in (E) represent 10 μm

Desmin is a structural and functional protein of pericytes that is important for the maintenance of vascular stability and pericyte contraction.^18^ The results of immunofluorescence assays showed that the protein expression of Desmin was significantly increased when EPCs were transfected with pAV‐Kir2.1 (Figure 3A). To observe the effect of transdifferentiated EPCs on the stability of vascular endothelial cells, the transformed EPCs were cocultured with RAECs in Matrigel matrix. Transdifferentiated EPCs exhibited longer vascular tubes in vitro (Figure 3BandC) and a higher capillary‐like structure maintenance rate (Figure 3D) than the control group, which indicates that Kir2.1 overexpression in EPCs can promote angiogenesis and stabilize neovascularization.

Typical and mature pericytes are easily regulated by local factors and induced by cell contraction. Therefore, we examined the ability of transdifferentiated EPCs to respond to NE stimulation. After a 1‐h treatment with NE, both cell bodies and nuclei of cells (GFP labelling) in the experimental group were wrinkled (Figure 3E, as shown by the arrow); however, in the control group, this effect was slower. Similarly, the collagen contraction experiment also confirmed this trend of results (Figure 3F), which showed that the EPC group overexpressing Kir2.1 exhibited higher contractile ability than the control group.

### 3.4 Transdifferentiation of EPCs into pericytes depends on the function of the Kir2.1 channel

Previous studies have shown that the function of the Kir2.1 protein in cell membranes depends on either its potassium channel transduction or its interactions with other protein molecules, though there is apparent cellular heterogeneity.^19^ Based on our previous research, we studied the mechanism of Kir2.1 channel function in EPC transdifferentiation by using the Kir2.1 channel blocker ML133 and the Kir2.1 agonist zacopride.

**FIGURE 4:**
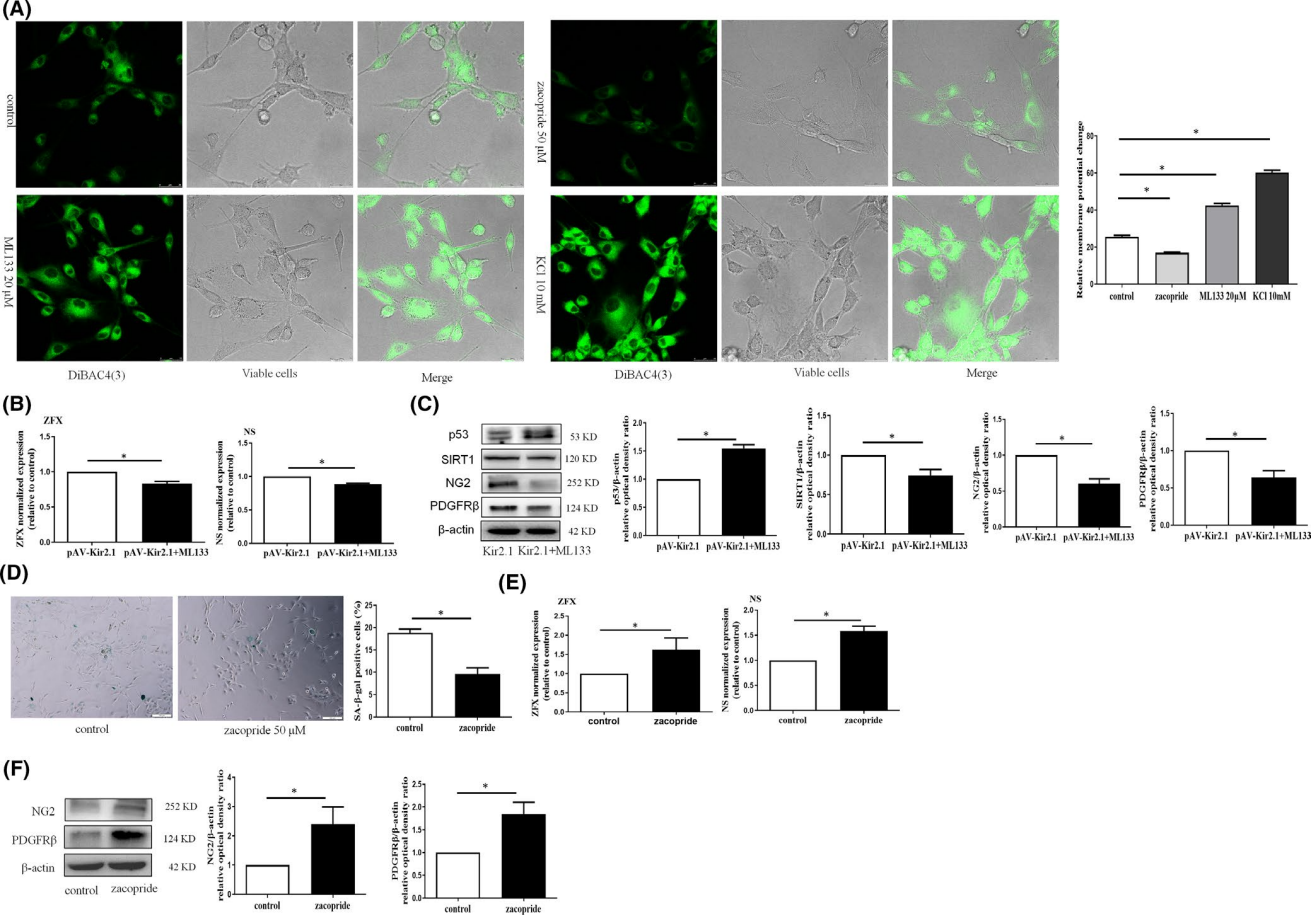
Transdifferentiation of EPCs into pericytes depends on the function of the Kir2.1 channel. (A) The cell resting membrane potential (RMP) was detected by DiBAC4(3) staining (5 µM, green). When the intracellular fluorescence intensity increased, the RMP increased. Conversely, a decrease in intracellular fluorescence intensity, that is a decrease in the RMP, indicated hyperpolarization. The relative fluorescence intensity was visualized by confocal laser microscope. (B) The stem cell stemness genes ZFX and NS in EPCs were detected by real‐time RT‐PCR. (C) The effects of ML133 on the senescence (p53 and SIRT1) and transdifferentiation (NG2 and PDGFRβ) of pAV‐Kir2.1‐transfected EPCs were detected by WB analysis. The relative optical densities of the bands in the gel were analyzed. (D) The senescence of EPCs in the control and zacopride pretreatment (working concentration, 50 µM) groups was detected with the SA‐β‐Gal method. (E) Alterations in the expression levels of genes related to stemness (ZFX and NS) and (F) transdifferentiation (NG2 and PDGFRβ) in zacopride‐pretreated EPCs were detected by WB analysis. The relative optical densities of the bands in the gel were analyzed (*p < 0.05). All values represent the mean ± SD for 3 separate experiments. Scale bars in (A) represent 25 μm, while those in (D) represent 100 μm

First, we observed the effect of the Kir2.1 channel on the RMP of EPCs with the membrane potential–sensitive probe DiBAC4(3). The Kir2.1 channel blocker ML133 enhanced DiBAC4(3) fluorescence staining (Figure 4A), suggesting that ML133 can cause EPC depolarization. In contrast, zacopride weakened DiBAC4(3) fluorescence staining, indicating that EPCs were hyperpolarized. Therefore, we investigated the mechanism of EPC transdifferentiation using the effects of both reagents on the Kir2.1 channel.

Subsequently, the treatment of Kir2.1‐overexpressing EPCs with ML133 was found to increase the expression of p53, NG2 and PDGFRβ; in contrast, ML133 inhibited the expression of ZFX, NS and SIRT1 (Figure 4BandC). In parallel, we incubated EPCs with the Kir2.1 agonist zacopride (50 µM) and found that it inhibited SA‐β‐Gal expression in EPCs (Figure 4D), suggesting that zacopride could reduce the incidence of cell senescence. Similarly, data from Figure 4EandF showed that zacopride could also promote the expression of ZFX, NS, NG2 and PDGFRβ in EPCs, suggesting that it promotes the maintenance of stemness and transformation of EPCs into a pericyte phenotype.

### 3.5 The slightly up‐regulated expression of Kir2.1 promotes EPC transdifferentiation into a pericyte phenotype by the Akt/mTOR/Snail signalling pathway

**FIGURE 5:**
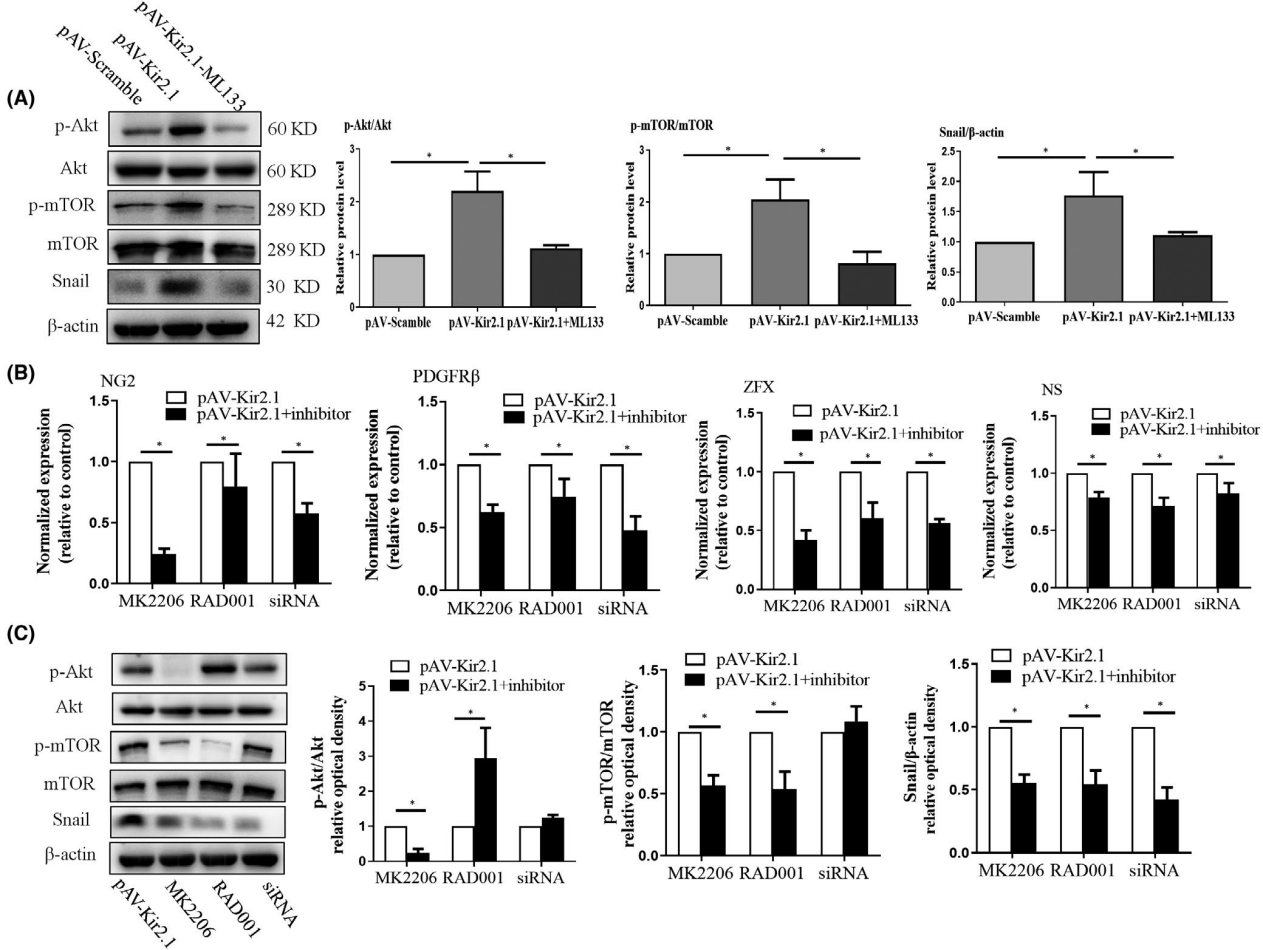
Slightly up‐regulated expression of Kir2.1 promotes EPC transdifferentiation into a pericyte phenotype by the Akt/mTOR/Snail signal pathway. (A) ML133 inhibited the up‐regulation of p‐Akt, p‐mTOR and Snail induced by pAV‐Kir2.1 transfection in EPCs. The relative optical densities of the bands in the gel were analyzed. (B) Inhibiting or blocking the activity or expression of the signalling molecules Akt, mTOR and Snail using the blockers MK2206 or RAD001 and Snail siRNA, respectively, reversed the biological effects of pAV‐Kir2.1 on the stemness and transdifferentiation of EPCs. (C) MK2206 attenuated the levels of the p‐Akt, p‐mTOR and Snail proteins in pAV‐Kir2.1‐transfected EPCs. RAD0001 increased the level of p‐Akt but decreased the levels of p‐mTOR and Snail in pAV‐Kir2.1‐transfected EPCs. Snail siRNA attenuated the level of Snail in pAV‐Kir2.1‐transfected EPCs but did not affect p‐Akt and p‐mTOR. All values represent the mean ± SD for 3 separate experiments. (*p < 0.05)

Based on the previous studies, we further investigated the role of potential molecular signalling pathways in Kir2.1‐mediated EPC transdifferentiation. We found that increased expression of Kir2.1 enhanced the level of intracellular Akt and mTOR phosphorylation and promoted expression of the Snail protein. However, there were no significant changes in the total Akt and mTOR levels compared with those in the control group. Accordingly, treatment with the Kir2.1 channel blocker ML133 abolished these effects (Figure 5A).

Next, the Akt phosphorylation blocker MK2206, the p‐mTOR inhibitor RAD001 and Snail siRNA were applied to inhibit or interfere with the expression of the corresponding signal molecules. Then, the effects of these changes on expression of the marker molecules NG2 and PDGFRβ and the stem stemness factors ZFX and NS were detected. The results showed that MK2206, RAD001 and Snail siRNA could attenuate the up‐regulated expression of NG2, PDGFRβ, NS and ZFX promoted by Kir2.1 (Figure 5B), suggesting that Akt/mTOR/Snail plays a central role in the EndoMT of Kir2.1‐induced EPCs.

Then, as expected, MK2206 was found to significantly inhibit Akt phosphorylation. MK2206 also inhibited mTOR phosphorylation and decreased the expression of Snail. Pretreatment with the mTOR blocker RAD001 reduced expression of the Snail protein. Surprisingly, we found that the mTOR inhibitor RAD001 significantly promoted Akt phosphorylation. However, intervention with Snail siRNA had no significant effect on the phosphorylation of Akt and mTOR (Figure 5C).

**FIGURE 6:**
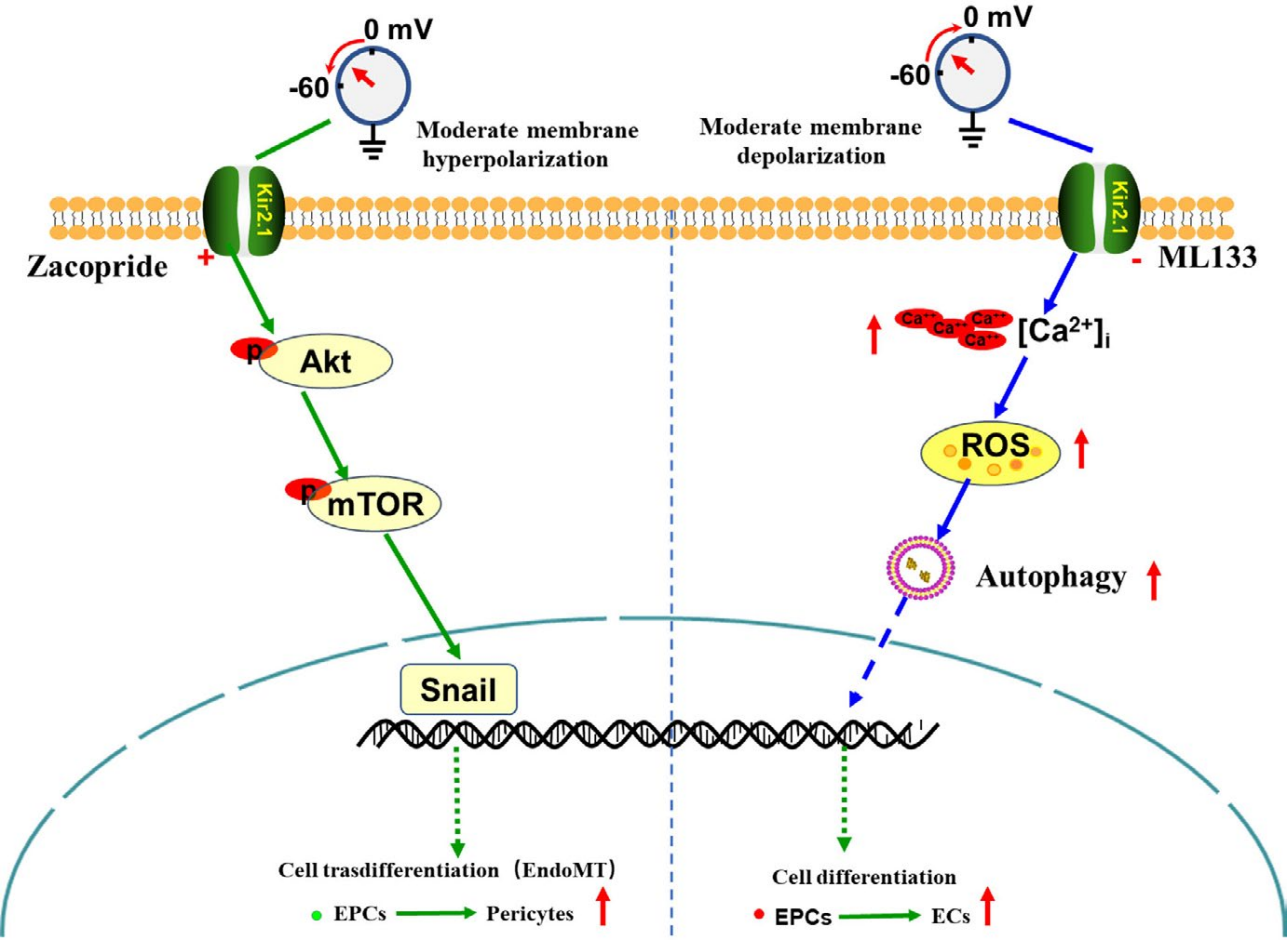
Diagrammatic representations of Kir2.1 channel–mediated regulation of the direction of EPC differentiation. After the Kir2.1 channel is inhibited, EPC depolarization is induced, and autophagy is then induced, promoting EPC differentiation into ECs. In contrast, if the Kir2.1 channel is agitated, EPC hyperpolarization is promoted, and the Akt/mTOR/Snail signalling pathway is excited, inducing the transdifferentiation of EPCs into mesenchymal cells (pericytes) and moderating/maintaining stem cell stemness

In summary, as shown in Figure 6, these results suggest that slight Kir2.1 overexpression could promote EPC transdifferentiation through the Akt/mTOR/Snail pathway.

## 4 DISCUSSION

To comprehensively investigate the role of the Kir2.1 channel in the development of EPCs, we first observed the effect of overexpression of Kir2.1 on EPC stemness and senescence, the essential biological characteristics of stem/progenitor cells. In this study, it was surprising to find that the slightly up‐regulated expression of Kir2.1, which was achieved by taking advantage of transfection with adenovirus at different MOI values, could promote the maintenance of stemness and reduce the senescence of EPCs. Subsequently, we found that Kir2.1 protein expression was also slightly increased under stimulation with reactive oxygen species (ROS) at a particular concentration. However, when ML133 blocked the function of the Kir2.1 channel, the apoptosis and senescence rate of EPCs was significantly increased in oxidative conditions (Figure S1 and 5). These data suggest that the Kir2.1 channel plays an essential role in response to changes in the surrounding microenvironment.

More researchers showed that CD44, CD31 and vWF were all related to the differentiation of EPCs into endothelial cells.^20^, ^21^, ^22^ Subsequently, we found that the expression of EPC‐related marker molecules CD44, CD31 and vWF decreased significantly after promoting the expression of EPC Kir2.1. For example, CD44 was involved in mediating the proliferation and activation of endothelial cells.^20^ These data suggest that changes in Kir2.1 expression may influence the differentiation or transdifferentiation process of EPCs. Traditionally, EPCs residing in the stem cell niche or bone marrow differentiate into vascular ECs, which play a vital role in repairing the endothelium of damaged blood vessels. However, Diez D et al.^23^ showed that under the influence of the local environment, especially specific concentrations of TGFβ1, EPCs obtain a mesenchymal phenotype, showing increased expression of the transcription factors slug, Snail, zeb1 and endothelin‐1. Therefore, the authors speculated that EPCs transdifferentiate into smooth muscle cell–like cells through an EndoMT‐like process. In this study, we found no change in the expression of TGFβ1 either in the gene expression of TGFβ1 or the protein level in the supernatant of culture medium after EPCs transfection. Silvia et al.^24^ suggested that EPC might be a source of cells with pericyte or perivascular mesenchymal phenotype and function. As reported in the literature, supportive vascular cells, such as pericytes and/or smooth muscle cells, may originate from endothelial cells themselves.^25^, ^26^

As reviewed by the first discoverer of EPCs, Asahara, endothelial cells and pericytes are homologous; namely, they are derived from vascular stem cells from the developmental perspective.^27^ Therefore, EndoMT may be an essential mechanism to recruit such pericytes during postnatal angiogenesis and neovascularization.^28^ Nevertheless, the molecular regulatory mechanisms involved in the process of EndoMT are mostly unknown.

As mentioned above, the Kir2.1 channel likely plays a crucial role in the response of EPCs to changes in the surrounding microenvironment. However, whether changes in Kir2.1 expression determine the process of EPC transdifferentiation is mainly unknown. In this study, we found that slightly elevated Kir2.1 expression inhibited EPC differentiation into ECs; in contrast, expression of the pericyte marker molecules NG2 and PDGFRβ was promoted, suggesting that the Kir2.1 channel contributes to the acquisition of a mesenchymal (pericyte) phenotype by EPCs.

Although NG2 and PDGFRβ are pericyte marker molecules, it must be noted that the levels of these markers in different tissues were highly variable. Therefore, pericyte‐related functions were also identified in transdifferentiated EPCs. Our data showed that the increased expression of Kir2.1 in EPCs cocultured with RAECs promoted in vitro angiogenesis and assisted in maintaining the stability of vessel tube formation. Further results showed that the cell contraction ability of EPCs transfected with Kir2.1 was significantly improved under stimulation with NE. Therefore, our data suggest that increased expression of Kir2.1 promotes EPC transdifferentiation into a pericyte phenotype.

The mechanism by which the Kir2.1 channel induces differentiation or transdifferentiation varies with the cell type. The possible mechanisms involved can be divided into two types: (1) the physiological channel functions of Kir2.1, which maintain the RMP (also called ion channel‐dependent functions) and (2) the membrane signal transduction pathway mediated by the Kir2.1 protein (ion channel–independent functions).^19^ The basic function of the Kir2.1 channel is to regulate the RMP of excitable and non‐excitable cells by regulating the permeability of the cell membrane to potassium ions. For rapidly proliferating stem cells or progenitors, the RMP is maintained at −20 mv to −40 mV by the Kir2.1 channel, while nerve cells remain in a relatively hyperpolarized state with an RMP of −60 mv to −80 mv.^19^, ^29^ A change in the RMP directly or indirectly affects the function of the corresponding cell.^29^, ^30^, ^31^ On the other hand, the Kir2.1 channel can also regulate cellular functions in an ion‐independent manner. According to researchers, Kir2.1 can be coupled with Stk38 to regulate the process of epithelial‐mesenchymal transition (EMT) and promote the invasion and metastasis of human gastric cancer cells.^19^ Leem et al. confirmed that Cdo promotes myogenic differentiation by regulating Kir2.1 activity through the phosphorylation of p38MAPK and MKK6 (EE).^32^

In this study, we first used the Kir2.1 channel blocker ML133 or the agonist zacopride to observe the role of the Kir2.1 channel in determining the RMP of EPCs through a rapid response fluorescence staining technique to determine the membrane potential. Compared with the blank control group, ML133 promoted the depolarization of EPCs. In contrast, the Kir2.1 agonist zacopride hyperpolarized EPCs. Further data showed that ML133 attenuated the effect of increased Kir2.1, which promoted the maintenance of EPC stemness and the transition of EPCs into a pericyte phenotype. Therefore, our data suggest that mediation of the ion permeation‐independent signalling pathway by the Kir2.1 channel is a key mechanism that enhances the stemness of EPCs and promotes EPC transdifferentiation into a pericyte phenotype.

Changes in the RMP, such as hyperpolarization or depolarization, trigger subsequent intracellular signalling pathways that change cellular behaviour.^33^ A study by Li et al.^34^ showed that enhancing Kir2.1 channel currents, which are related to the regulatory mechanism of the Akt/PI3‐kinase activity signalling pathway, rescued acute ischaemic arrhythmia triggered by hypoxia‐induced RMP depolarization. Ilaria et al.^30^ showed that membrane hyperpolarization inhibited Wnt signalling, thus promoting the development of daughter neurons. Therefore, some researchers have also confirmed that cell membrane hyperpolarization plays a key role through Notch, Ca^2+^ sparks, or the PKC signalling pathway and speculated that the specific signalling mechanism is closely related to the factors and type of cells stimulated.^35^, ^36^, ^37^ This study found that increasing Kir2.1 expression could promote the phosphorylation of both Akt and mTOR. It was also found that blocking Akt phosphorylation by MK2206 could effectively reduce the degree of mTOR phosphorylation. Further study showed that the inhibition of Akt and mTOR phosphorylation by the inhibitors MK2206 and RAD001, respectively, reduced the Kir2.1‐induced phenotypic transformation of EPCs to a pericyte phenotype, suggesting that Kir2.1‐induced changes in the RMP can affect the development of EPC EndoMT through the Akt/mTOR pathway. Surprisingly, although RAD001 significantly blocked the mTOR phosphorylation process, it also paradoxically promoted Akt phosphorylation. Some literature confirmed that RAD001‐induced p‐Akt up‐regulation was due to the autocrine release of insulin‐like growth factor‐1 (IGF‐1) with the subsequent activation of the IGF‐1 receptor (IGF1R) in cell lines.^38^, ^39^, ^40^ So we speculated that this RAD001‐mediated p‐Akt up‐regulation was concerned with the IGF‐1/IGF‐1 receptor autocrine loop: (1) IGF1 and IGF1R are expressed in EPCs derived from rat bone marrow, which according to the microarray data accessed at GEO database with accession number GSE49510 (Figure S6); (2) it has been confirmed that EPCs can secrete IGF‐1 and express IGF1R, which serves the vital cell biological function,^41^, ^42^, ^43^ but it requires further exploration.

Some studies suggest that Snail is a pivotal transcription factor involved in EndoMT and EMT.^44^, ^45^ In this study, the slight up‐regulation of Kir2.1 was found to promote Akt/mTOR phosphorylation, which promoted the expression of Snail in EPCs. As shown in Figure 5, once the expression of Snail was reduced by siRNA interference, the effect of Kir2.1 on promoting EPC EndoMT was weakened, which suggests that the Akt/mTOR/Snail pathway is involved in the process of Kir2.1‐mediated EPC transformation to a pericyte phenotype.

In conclusion, combined with our previous studies, our results have demonstrated the following findings (shown in Figure 6). Therefore, we speculate that the Kir2.1 channel is likely a switch molecule by which EPCs perceive the stem niche and thus determine the direction of EPC differentiation or transdifferentiation, and Kir2.1 may be a new therapeutic target for vascular remodelling diseases.

## CONFLICT OF INTEREST

All authors of this article declare that there is no conflict of interest.

## AUTHOR CONTRIBUTIONS

**Xiaodong Cui:** Data curation (equal); Formal analysis (equal); Funding acquisition (equal); Writing‐original draft (equal); Writing‐review & editing (equal). **Xiaoxia Li:** Data curation (equal); Formal analysis (equal); Writing‐review & editing (equal). **Yanting He:** Investigation (equal); Methodology (equal). **Jie Yu:** Investigation (equal); Methodology (equal). **Naijun Dong:** Investigation (equal); Methodology (equal). **Robert Chunhua Zhao:** Conceptualization (lead); Funding acquisition (lead); Project administration (lead).

## Supporting information

Fig S1‐S6

## Data Availability

The data that support the findings of this study are available from the corresponding author upon reasonable request.
