# Supplementary material for: Slight up‐regulation of Kir2.1 channel promotes endothelial progenitor cells to transdifferentiate into a pericyte phenotype by Akt/mTOR/Snail pathway
Source: J Cell Mol Med. 2021 Sep 30;25(21):10088–100. doi: 10.1111/jcmm.16944 (PMC8572793; doi:10.1111/jcmm.16944)
Supplement: Supplementary file 1 — Fig S1‐S6 [file JCMM-25-10088-s001.docx]

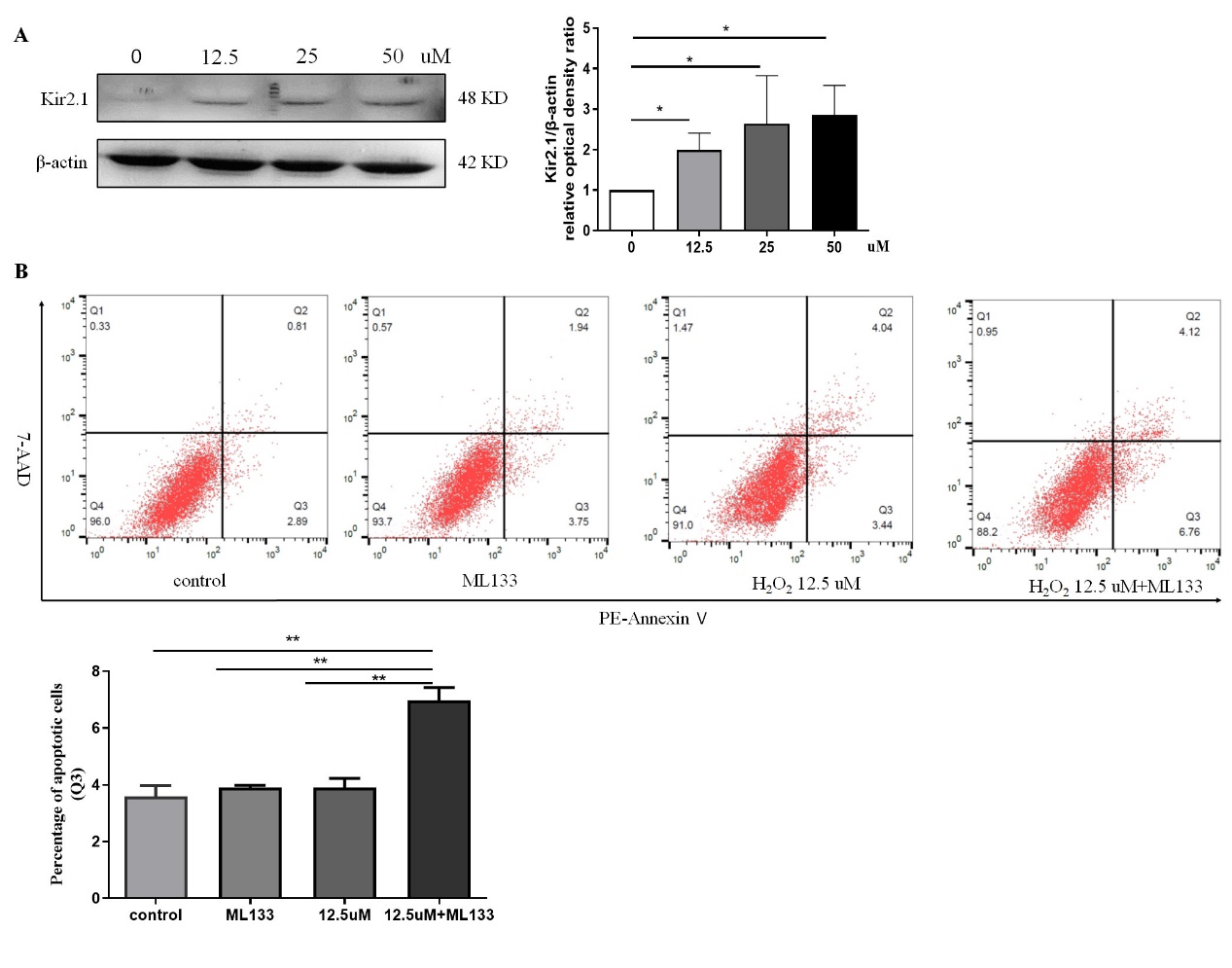


**Supplemental Figure 1** The expression of Kir2.1 was slightly increased under stimulation with H_2_O_2_ at a various concentration. (A) The expression of Kir2.1 was detected by WB analysis. (B) However, when ML133 blocked the function of the Kir2.1 channel, the senescence rate of EPCs was significantly increased. All values represent the mean±SD for 3 separate experiments. (^*^*P*<0.05, ^**^*P*<0.01)

**
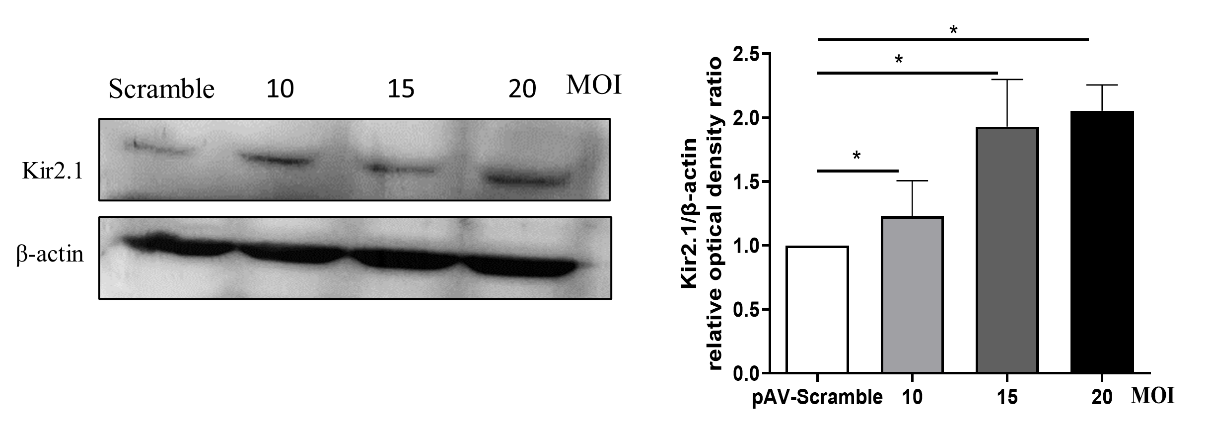
**

**Supplemental Figure 2** Kir2.1 protein expression was detected by WB. Relative data are used to represent the Kir2.1 protein expression in EPCs transfected with adenovirus at different MOIs. All values represent the mean±SD for 3 separate experiments. (^*^*P*<0.05)


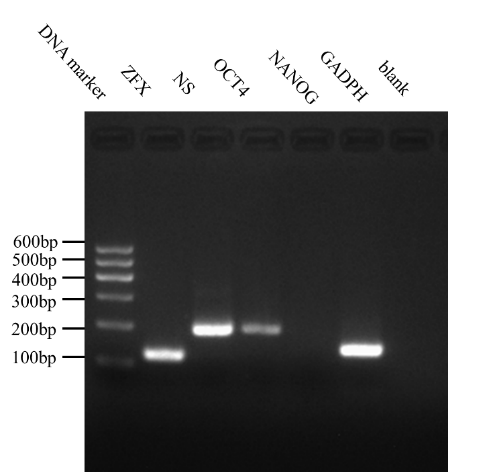


**Supplemental Figure 3** RT-PCR analysis of the expression of stemness markers in EPCs at 3th passage. As it is evident, EPCs express ZFX and NS, but lower in OCT4 and NANOG genes. GAPDH was used as an internal control.


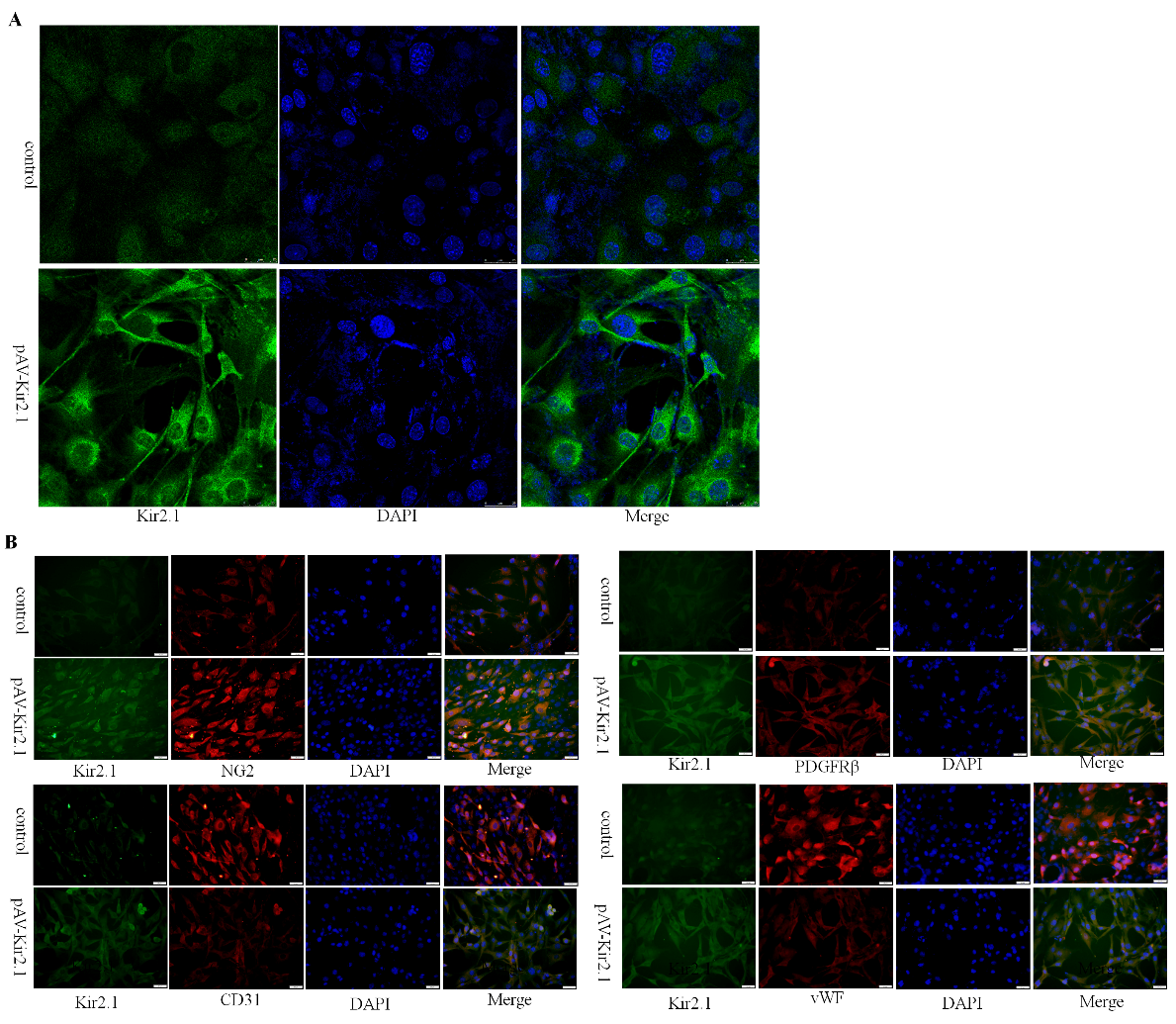


**Supplemental Figure 4** The association between Kir2.1 overexpression and endothelial cell marker molecules CD31 and vWF by immunofluorescence staining. (A) the vexpression of Kir2.1 were visualized by confocal laser microscope. (B) Endothelial cell marker molecules CD31 and vWF, pericyte marker NG2 and PDGFRβ, were performed by tripledouble immunofluorescence staining with antibodies and DAPI solution. The fluorescence intensity of protein expression were visualized by [fluorescence](javascript:;) [microscope](javascript:;).


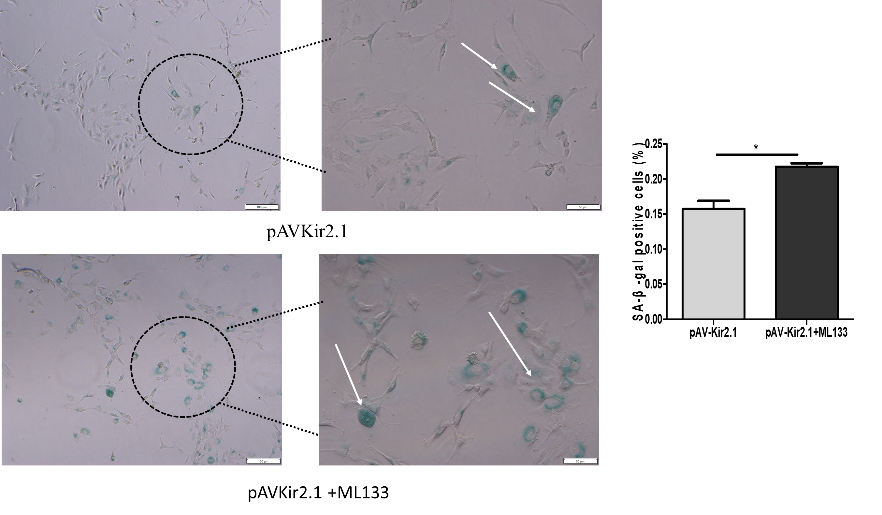


**Supplemental Figure 5** The effects of ML133 on the senescence were detected by the SA-β-Gal method. EPC senescence was detected using the SA-β-Gal method (**P* < 0.05). Scale bars in represent 100 μm and 50 μm.

**Supplemental Figure 6** IGF1 and IGF1R are expressed in EPCs derived rat bone marrow, which according to the microarray data accessed at GEO database with accession number GSE49510. Samples 1-4, respectively labeled as GSM1200243, GSM1200244, GSM1200245 and GSM1200246, which represented culture-induced EPCs by inoculation of bone marrow mononuclear cells with high density and regular density culture.
